# Efficacy and safety of TACE/HAIC combined with targeted immunotherapy versus targeted immunotherapy for advanced hepatocellular carcinoma: a meta-analysis

**DOI:** 10.3389/fonc.2025.1669460

**Published:** 2025-11-19

**Authors:** Yuting Liao, Wenliang Zhu, Jianquan Zhang

**Affiliations:** 1 Department of Hepatobiliary Surgery, Central South University Xiangya Medical College Affiliated Haikou Hospital, Haikou, Hainan, China; 2 Department of Intervention, Guangxi Medical University Cancer Hospital, Nanning, Guangxi, China

**Keywords:** hepatocellular carcinoma, targeted therapy, immunotherapy, triple therapy, dual therapy

## Abstract

**Introduction:**

The purpose of this study was to investigate the efficacy and safety of triple therapy with transcatheter arterial chemoembolization (TACE)/hepatic arterial infusion chemotherapy (HAIC) in patients with advanced hepatocellular carcinoma (HCC).

**Methods:**

A systematic literature search of multiple databases ending with publications from May 2024 was conducted. Interventions with TACE/HAIC combined with targeted immunotherapy versus targeted immunotherapy and patients with advanced HCC were included in this meta-analysis. Data from the 13 included studies, encompassing 2, 662 patients with HCC, were pooled using fixed- or random-effects models. The results are reported as hazard ratios (HRs) or risk ratios (RRs) with corresponding 95% confidence intervals (CIs).

**Results:**

In contrast to targeted immunotherapy, TACE/HAIC combined targeted immunotherapy can significantly improve overall survival (OS), progression-free survival (PFS) the disease control rate (DCR), and the objective response rate (ORR) in patients with advanced HCC. At the same time, the overall incidence of treatment-related adverse events (AEs) and treatment-related AEs of grade 3 or greater also increased, but only a few of the specific treatment-related AEs increased.

**Conclusions:**

Given that combined TACE/HAIC with targeted immunotherapy significantly improves patient OS, PFS, DCR, and ORR, it may become standard for the treatment of patients with advanced HCC.

## 1 Introduction

Primary liver cancer ranks as the sixth most common malignant tumor globally. Due to its large population base and high disease incidence, China bears the highest number of liver cancer cases worldwide (1). Hepatocellular carcinoma (HCC) accounts for approximately 90% of primary liver cancers, representing the predominant pathological type (2).

The management of advanced HCC has evolved into a comprehensive strategy integrating locoregional and systemic therapies (3). Key modalities include targeted therapy, immunotherapy, and locoregional interventions such as Transcatheter Arterial Chemoembolization (TACE) and Hepatic Arterial Infusion Chemotherapy (HAIC). Targeted therapy, centered on anti-angiogenic agents and multi-target tyrosine kinase inhibitors (TKIs), is well-established. Based on evidence from phase III clinical trials, agents including bevacizumab, ramucirumab, sorafenib, lenvatinib, regorafenib, and cabozantinib have received FDA approval for clinical use (4). Among locoregional approaches, TACE—which involves the intra-arterial delivery of chemotherapeutic agents followed by vascular embolization to occlude tumor-feeding arteries and induce ischemic necrosis—remains a first-line standard for intermediate- to advanced-stage HCC (5). HAIC, characterized by continuous intra-arterial infusion of chemotherapy, achieves high local drug concentrations. Studies have confirmed its efficacy in significantly prolonging disease-free survival when used as sequential post-operative therapy in HCC patients with microvascular invasion (6).

In recent years, immunotherapy has emerged as a cornerstone of systemic treatment for advanced HCC, often combined with targeted agents (7). Although dual-combination regimens, such as “immunotherapy plus targeted therapy” or “dual immunotherapy, “ have demonstrated improved survival outcomes in multiple studies (8–10), the five-year survival rate for advanced HCC patients remains below 20%, indicating a persistently poor prognosis (11, 12). Against this backdrop, triple-therapy strategies combining locoregional intervention (TACE/HAIC) with targeted and immunotherapy have gained significant research interest. Preliminary data suggest these regimens can substantially improve tumor response rates and conversion-to-surgery rates, with a manageable safety profile (13–15). Mechanistically, the synergy may arise from enhanced tumor immunogenicity, promotion of immune-mediated tumor killing, and prolonged immunologic memory, ultimately achieving synergistic local and systemic disease control (16).

However, high-quality clinical evidence regarding TACE/HAIC combined with targeted and immunotherapy remains limited. On one hand, there is insufficient head-to-head evidence comparing this triple-therapy approach against dual therapy (targeted plus immunotherapy) alone. On the other hand, while TACE and HAIC differ fundamentally in their mechanisms—TACE combining chemotherapy with embolization versus HAIC relying on sustained chemotherapeutic infusion—their comparative efficacy and safety profiles when integrated with targeted and immunotherapy have not been systematically elucidated.

Therefore, this meta-analysis aims to systematically evaluate the benefits of TACE/HAIC combined with targeted and immunotherapy over dual therapy alone, and to clarify the differential impact of TACE versus HAIC on efficacy and safety outcomes in patients with advanced HCC. The findings are expected to provide more robust evidence to guide individualized clinical application of this combination strategy.

## 2 Methods

### 2.1 Search strategy

An extensive collection of literature published up to May 2024 was searched through PubMed, Embase, Web of Science, and the Cochrane Library. The search words included keywords and free words related to hepatocellular carcinoma, targeted therapy, and immunotherapy, such as “hepatocellular carcinoma”, “programmed cell death protein 1 (PD-1) inhibitors”, “programmed cell death 1 ligand 1(PD-L1) inhibitors”, “targeted therapy”, “hepatic arterial infusion chemotherapy (HAIC)” and “ transcatheter arterial chemoembolization (TACE)”. In addition, the studies or reviews are manually searched to avoid missing potential studies. No language restrictions were set during the retrieval process.

### 2.2 Inclusion and exclusion criteria

#### 2.2.1 Inclusion criteria

1) cohort or case cohort studies or randomized controlled trial (RCT) experiments; 2) the purpose of the studies was to evaluate the efficacy and safety of TACE/HAIC combined with targeted immunotherapy and targeted immunotherapy in patients with BCLC stage B or C HCC; 3) the results included at least one of the following: overall survival (OS), progression-free survival (PFS), objective response rate (ORR), disease control rate (DCR), and adverse events (AEs).

#### 2.2.2 Exclusion criteria

1) case report, case series, comments, review, meta-analysis, and other literature types; 2) literature with repeated and overlapping research data; 3) the study did not conform to the purpose; and 4) the full text was not available.

### 2.3 Data extraction and quality assessment

Literature quality evaluation and data extraction of the included literature were performed using uniform criteria by two researchers independently. In cases of disagreement, the researchers discussed the study together or consulted with a third researcher. RCT experiments were evaluated using The Cochrane Collaboration’s Risk of Bias Tool, and cohort studies were evaluated using the Newcastle-Ottawa Scale (NOS).

Data extraction included two parts: 1) basic information of the studies and the participants, including gender, age, and etc; and 2) interventions (TACE/HAIC combined with targeted immunotherapy as treatment group and targeted immunotherapy as control group) and results.

### 2.4 Statistical analysis

All statistical analyses in this study were performed using Stata 18.0 software.

#### 2.4.1 Selection and pooling of effect measures

For time-to-event outcomes such as OS and PFS, hazard ratios (HRs) with 95% confidence intervals (CIs) were used as effect measures. For dichotomous outcomes, relative risk (RR) with 95% CIs was applied. When available, these values were directly extracted from the original studies.

#### 2.4.2 Heterogeneity testing and model selection

Heterogeneity among studies was assessed using the Cochrane Q test and the I² statistic. I² values of 25%, 50%, and 75% were considered indicative of low, moderate, and high heterogeneity, respectively. A fixed-effects model was used when I² < 50%; otherwise, a random-effects model was applied.

#### 2.4.3 Subgroup and sensitivity analyses

To explore potential sources of heterogeneity, a subgroup analysis was conducted based on the type of locoregional therapy (TACE vs. HAIC) to evaluate its impact on outcomes and heterogeneity. A leave-one-out sensitivity analysis was performed; if the exclusion of a single study resulted in a reduction of heterogeneity from significant (I² ≥ 50%) to non-significant (I² < 50%), that study was considered a major source of heterogeneity and excluded.

#### 2.4.4 Assessment of publication bias

Publication bias was evaluated using funnel plots and Begg’s test. If bias was suggested but all studies fell within the 95% CI region of the funnel plot, the trim-and-fill method was applied for adjustment. Otherwise, apparent outliers were removed before re-analysis.

A two-sided P-value < 0.05 was considered statistically significant.

## 3 Result

### 3.1 Study selection

A total of 2, 358 records were initially identified through searches of PubMed, Cochrane, Embase, and Web of Science. After removing 513 duplicates, 1, 827 records were excluded based on title and abstract screening. Following full-text retrieval and assessment, five articles were excluded due to unavailability of the full text, resulting in 13 articles being included in the final meta-analysis (**Figure S1**).

### 3.2 Study characteristics

The specific characteristics are shown in **Table 1**. The 13 reports (17–29) were published from 2021–2024. A total of 3098 original participants were included, where (17, 18, 23, 28, 29) used propensity score matching (PSM) to reduce confounding bias (18, 19), used stabilized inverse probability of treatment weighting (iIPTW), and 2664 patients were included in this study. All the studies were retrospective studies. The majority of the patients were middle-aged and elderly people, and most were male. Of these patients, according to BCLC scoring, there were no stage A patients, 16.5% were stage B, and 83.5% were stage C. The intervention factors in all experimental groups included HAIC or TACE, Immune checkpoint inhibitors, and targeted agents. The control groups received Immune checkpoint inhibitors combined with the targeted drugs. Each study had a NOS score of greater than or equal to 6 points, indicating higher study quality (**Table 2**).

**Table 1:** Baseline characteristics of patients in the trials included in the meta-analysis.

a							
Authors	Yeas	interventions					Participants
Huang JT (14)	2022	TACE+sorafenib + camrelizumab / lenvatinib + sintinimab					24
		sorafenib + camrelizumab /lenvatinib+sintinimab					24
Wang J (15)	2023	TACE+Lenvatinib+pembrolizumab/camrelizumab/sintilimab					43
		Lenvatinib+pembrolizumab/camrelizumab/sintilimab					43
Xin Y (16)	2023	TACE+Lenvatinib+sintilimab/tislelizumab/camrelizumab					60
		Lenvatinib+sintilimab/tislelizumab/camrelizumab					58
Yin YL (17)	2023	TACE+ sorafenib /lenvatinib +camrelizumab +					28
		sorafenib /lenvatinib+camrelizumab +					16
Jin ZC (18)	2024	TACE + ICIs+anti-VEGF antibody					805
		ICIs+anti-VEGF antibody					437
Zhang JX a (19)	2024	TACE+lenvatinib/sorafenib+camrelizumab/sintilimab/atezolizumab					106
		lenvatinib/sorafenib+camrelizumab/sintilimab/atezolizumab					109
Zhang JX b (20)	2024	TACE+sorafenib/lenvatinib+ camrelizumab/ sintilimab					54
		sorafenib/lenvatinib+camrelizumab/ sintilimab					54
Chen S (21)	2021	HAIC+lenvatinib+Pembrolizumab					84
		lenvatinib+Pembrolizumab					86
Mei J (22)	2021	HAIC+lenvatinib+PD-1 inhibitors					45
		lenvatinib+PD-1 inhibitors					25
Chang X (23)	2024	HAIC+lenvatinib+PD-1 inhibitors					103
		lenvatinib+PD-1 inhibitors					61
Diao L (24)	2024	HAIC+lenvatinib+PD-1 inhibitors					58
		lenvatinib+PD-1 inhibitors					63
Guan R (25)	2024	HAIC+lenvatinib+PD-1 inhibitors					55
		lenvatinib+PD-1 inhibitors					55
Li YY (26)	2024	HAIC+rivoceranib+camrelizumab					83
		Rivoceranib+camrelizumab					83

b							
Authors	Male/ Female	age(mean±SD)	ECOG-P(0/1/2)	BCLC(B/C)	Liver cirrhosis(Y/N)	tumour thrombus (Y/N)	Extrahepatic metastasis (Y/N)
Huang JT (14)	20/4	58.0±10.7	11/13/0	0/24	–	–	9/15
	21/3	56.5±14.0	9/15/0	0/24	–	–	13/11
Wang J (15)	38/5	57.0±10.53	16/27/0	8/35	38/5	–	22/21
	37/6	58.0±10.52	14/29/0	7/36	35/8	–	25/18
Xin Y (16)	54/6	57.5	55/5/0	23/35	–	28/32	18/42
	51/7	54.5	53/5/0	21/39	–	17/41	26/32
Yin YL (17)	22/6	54	7/15/6	4/24	–	16/12	16/12
	13/3	62.5	1/12/2	3/13	–	10/6	10/6
Jin ZC (18)	693/ 112	54±3.75	464/308/33	–	593/221	–	471/334
	379/ 59	56±3.75	257/162/18	–	325/112	–	258/179
Zhang JX a (19)	12/94	–	65/41/0	0/106	–	106/0	28/78
	11/98	–	66/43/0	0/109	–	109/0	29/80
Zhang JX b (20)	46/8	–	43/11/0	23/31	–	–	19/35
	47/7	–	41/13/0	21/33	–	–	20/34
Chen S (21)	72/12	52 ±6.25	38/46/0	22/62	57/27	49/35	20/64
	71/15	53±6.25	35/51/0	21/65	58/28	55/31	24/62
Mei J (22)	38/7	49.1± 10.6	–	5/40	40/5	36/9	15/30
	18/7	50.1± 12.3	–	3/22	18/7	18/7	13/12
Chang X (23)	91/12	52.0±8.82	–	4/99	–	–	32/71
	57/4	56.0±7.88	–	2/59	–	–	20/41
Diao L (24)	49/9	–	–	24/34	36/22	32/26	19/39
	50/13	–	–	25/38	37/26	30/33	21/42
Guan R (25)	49/6	53.6±11.44	–	0/55	35/20	/20	–
	48/7	54.2±9.95	–	0/55	36/19	/22	–
Li YY (26)	76/7	–	81/2/0	0/83	–	0/83	51/32
	77/6	–	82/1/0	0/83	–	0/83	41/42

TACE, Transcatheter arterial chemoembolization; HAIC, hepatic arterial chemoembolization; ICIs, immune checkpoint inhibitors; anti-VEGF, anti-vascular endothelial growth factor; TKIs, tyrosine kinase inhibitors; PD-1, PD-1, programmed death-1; SD, standard deviation; BCLC, Barcelona Clinic Liver Cancer; ECOG, Eastern Cooperative Oncology Group; Y, yes; N, No.

**Table 2:** NOS quality evaluation.

NO.	Authors	Years	NOS			
			Selection	Comparability	Outcome	Scores
1	Huan JT (14)	2022	⭐⭐⭐⭐	⭐⭐	⭐⭐	8
2	Wang J (15)	2023	⭐⭐⭐⭐	⭐⭐	⭐⭐	8
3	Xin Y (16)	2023	⭐⭐⭐⭐	⭐⭐	⭐⭐	8
4	Yin YL (17)	2023	⭐⭐⭐⭐	⭐⭐	⭐⭐	8
5	Jin ZC (18)	2023	⭐⭐⭐⭐	⭐⭐	⭐⭐	8
6	Zhang JX a (19)	2024	⭐⭐	⭐⭐	⭐⭐	6
7	Zhang JX b (20)	2024	⭐⭐	⭐⭐	⭐⭐	6
8	Chen S (21)	2021	⭐⭐⭐⭐	⭐⭐	⭐⭐	8
9	Mei J (22)	2021	⭐⭐⭐⭐	⭐⭐	⭐⭐	7
10	Chang X (23)	2024	⭐⭐⭐⭐	⭐⭐	⭐⭐	8
11	Diao L (24)	2024	⭐⭐⭐⭐	⭐⭐	⭐⭐	7
12	Guan R (25)	2024	⭐⭐⭐⭐	⭐	⭐	6
13	Li YY (26)	2024	⭐⭐⭐⭐	⭐⭐	⭐⭐	8

### 3.3 OS

**Figure 1:**
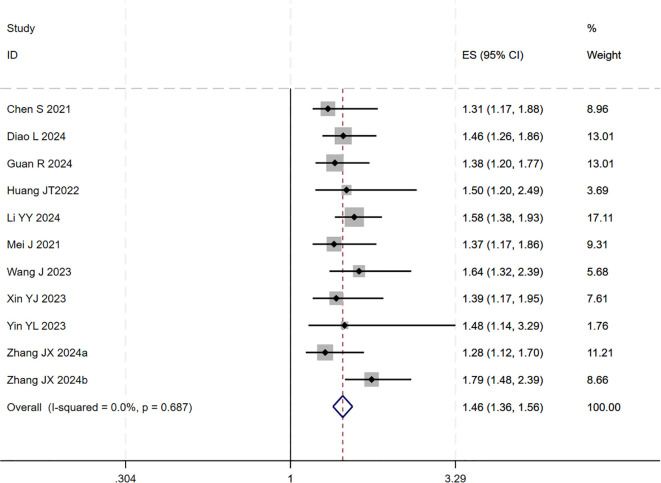
The forest plot illustrates the impact of TACE/HAIC combined with targeted immunotherapy on OS. Results demonstrate that the combination significantly improves OS compared to targeted immunotherapy alone. Squares represent HRs, horizontal lines indicate 95% CIs, and the diamond shows the pooled HR with its 95% CI. No significant heterogeneity was observed among studies (I² = 0.0%, p = 0.687). The overall pooled effect size was 1.46 (95% CI: 1.36, 1.56).

**Figure 2:**
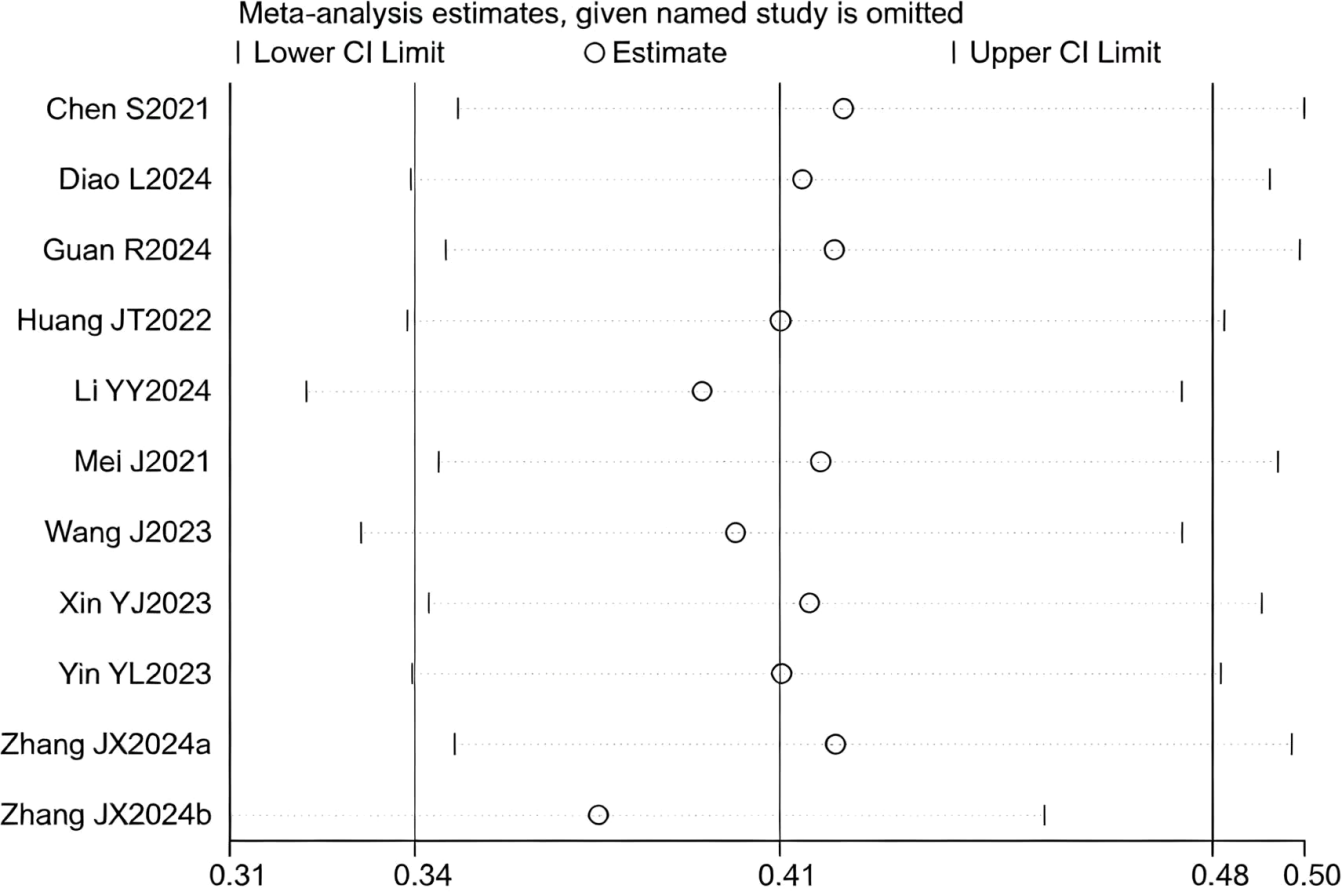
Heterogeneity test of the OS results from the included studies. The sensitivity analysis revealed that there was no significant heterogeneity between the studies. Each circle on the vertical axis represents the exclusion of one experiment, and the short vertical lines at the two side ends represent the corresponding 95%CIs. From left to right, the three vertical lines represent the lower limit of the 95%CI, the mean, and the upper limit of the 95%CI for the overall effect.

12 studies (17–25, 27–29) reported HR for OS. The initial pooled analysis indicated significant heterogeneity among these studies. To ensure the accuracy and robustness of the findings, subgroup and sensitivity analyses were performed, which identified the study by (21) as the primary source of heterogeneity. After excluding this study, the heterogeneity test showed no residual heterogeneity among the remaining studies (I² = 0.0%, p = 0.687). The pooled effect size demonstrated that the triple therapy (TACE/HAIC combined with targeted and immunotherapy) significantly improved OS compared to dual therapy (targeted plus immunotherapy) alone (HR=1.46, 95% CI: 1.36 to 1.56, P < 0.001) (**Figure 1**). Sensitivity analysis confirmed the robustness of this result (**Figure 2**).

**Figure 3:**
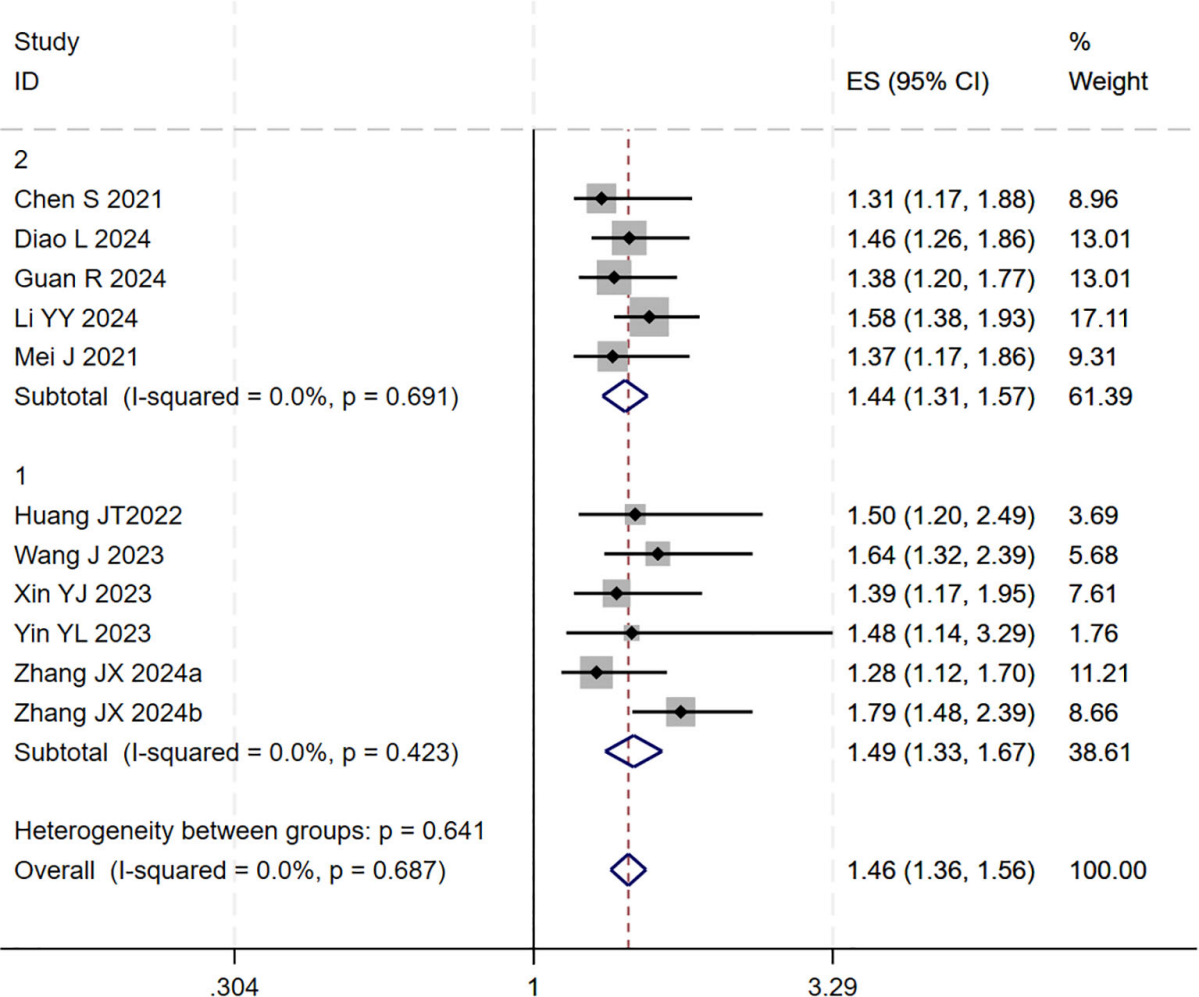
The forest plot shows subgroup analysis of OS by local therapy (Subgroup 1: TACE; Subgroup 2: HAIC). No significant difference in OS was found between subgroups (p=0.641). Squares represent HRs, horizontal lines show 95% CIs, and diamonds indicate pooled HRs. Both subgroups showed no heterogeneity (I²=0.0%) with pooled HRs of 1.49 (1.33-1.67) for TACE and 1.44 (1.31-1.57) for HAIC. Overall pooled HR was 1.46 (1.36-1.56) with no significant heterogeneity (I²=0.0%, p=0.687).

A subsequent subgroup analysis was conducted (**Figure 3**). The results indicated that the triple therapy significantly improved OS regardless of whether TACE or HAIC was used as the locoregional modality (P < 0.001 for both subgroups). The test for interaction between the two subgroups showed no statistically significant difference (p = 0.641), suggesting that the type of locoregional therapy did not substantially influence the overall survival benefit of the triple-therapy approach.

Publication bias was assessed using a funnel plot and Egger’s test. Egger’s test yielded a p-value of 0.161 (p >0.05), indicating no significant evidence of publication bias in this analysis (**Figure S2**).

### 3.4 PFS

Ten studies (18–21, 23–25, 27–29) reported HR for PFS. The initial pooled analysis revealed substantial heterogeneity among the studies. To assess the robustness of the findings, subgroup and sensitivity analyses were performed, which identified the study by (21) as the primary source of heterogeneity. After excluding this study, heterogeneity was significantly reduced. The pooled effect size demonstrated that the triple therapy significantly improved PFS compared to dual therapy.

Subsequent subgroup analysis based on the type of locoregional therapy (TACE vs. HAIC) indicated that triple therapy significantly prolonged PFS regardless of the approach used (p < 0.001 for both subgroups). The test for interaction showed no statistically significant difference between the subgroups (p = 0.476), suggesting that the choice of locoregional modality did not significantly influence the PFS benefit.

**Figure 4:**
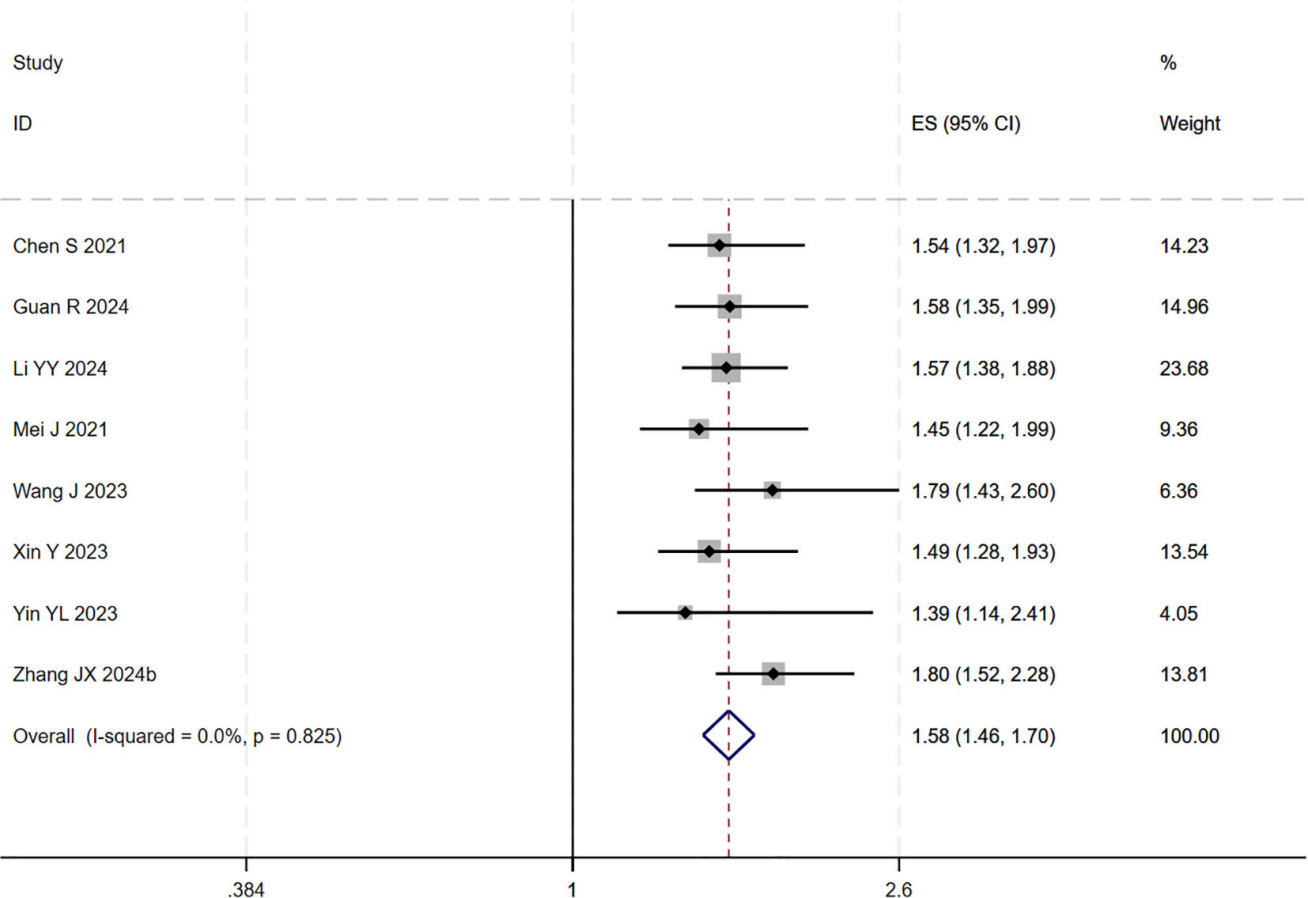
The forest plot demonstrates the effect of TACE/HAIC combined with targeted immunotherapy on PFS. Results indicate the combination significantly improves PFS compared to targeted immunotherapy alone. Squares represent HRs, horizontal lines indicate 95% CIs, and the diamond shows the pooled HR with its 95% CI. No significant heterogeneity was observed among studies (I² = 0.0%, p = 0.825). The overall pooled effect size was 1.58 (95% CI: 1.46, 1.70).

**Figure 5:**
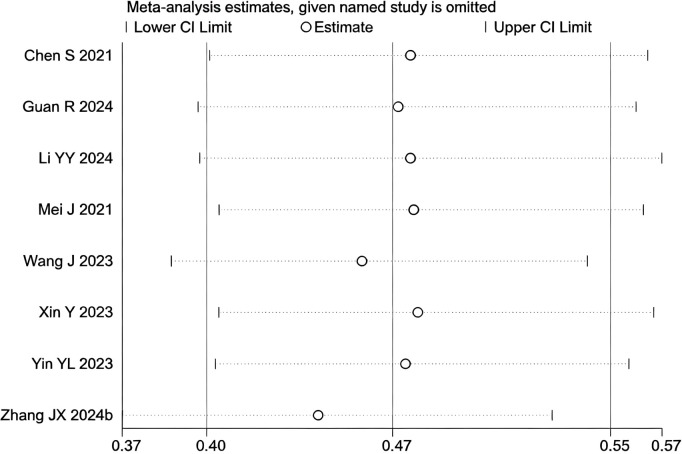
Heterogeneity test of the PFS results from the included studies. The sensitivity analysis revealed that there was no significant heterogeneity between the studies. Each circle on the vertical axis represents the exclusion of one experiment, and the short vertical lines at the two side ends represent the corresponding 95%CIs. From left to right, the three vertical lines represent the lower limit of the 95%CI, the mean, and the upper limit of the 95%CI for the overall effect.

**Figure 6:**
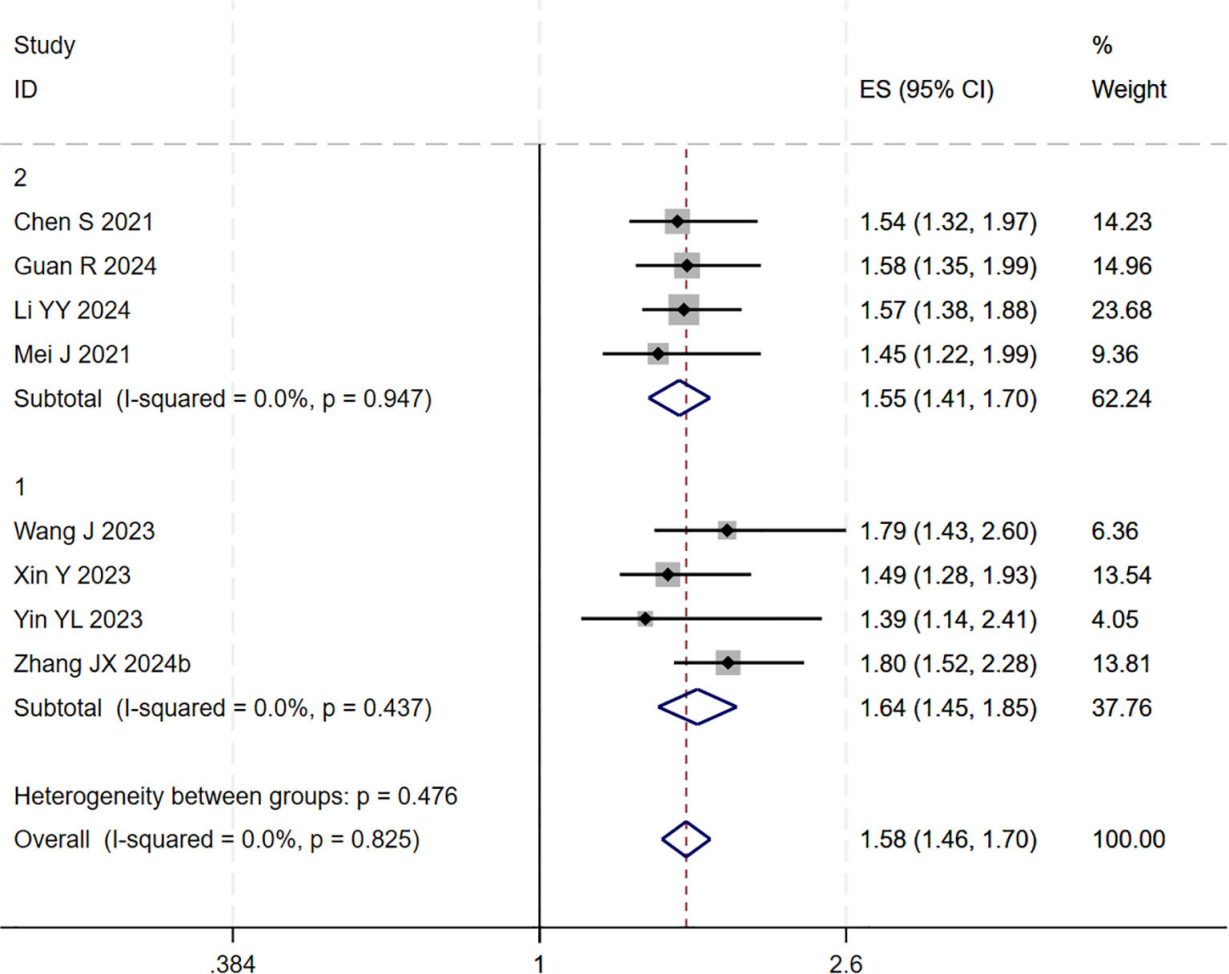
The forest plot presents a subgroup analysis of PFS based on local therapy (Subgroup 1: TACE; Subgroup 2: HAIC). No statistically significant difference in PFS was observed between the subgroups (p=0.476). Squares represent individual HRs, horizontal lines indicate 95% CIs, and diamonds denote pooled HRs. Both subgroups showed no heterogeneity (I²=0.0%) with pooled HRs of 1.64 (1.45-1.85) for TACE and 1.55 (1.41-1.70) for HAIC. The overall pooled HR was 1.58 (1.46-1.70) with no significant heterogeneity (I²=0.0%, p=0.825).

During the assessment for publication bias, one study was identified as falling outside the confidence interval. Considering the preceding analyses, it was postulated that (27) might introduce substantial bias. After excluding the study, heterogeneity analysis showed a further reduction (I² = 0.0%, p = 0.825). Sensitivity analysis did not identify any other studies with a significant influence on the results (**Figures 4**, **5**). The analysis was repeated, and the results remained consistent: triple regimen significantly improved PFS (HR=1.58, 95% CI: 1.46–1.70, p < 0.001). Both the TACE and HAIC subgroups continued to show significant PFS improvement (p < 0.001), with the interaction test remaining non-significant (p = 0.476) (**Figure 6**), further supporting the conclusion that the type of locoregional therapy has a limited impact on efficacy.

Publication bias was assessed using a funnel plot and Egger’s test. The result of Egger’s test (p = 0.113 > 0.05) indicated no significant evidence of publication bias in this analysis (**Figure S3**).

### 3.5 DCR

**Figure 7:**
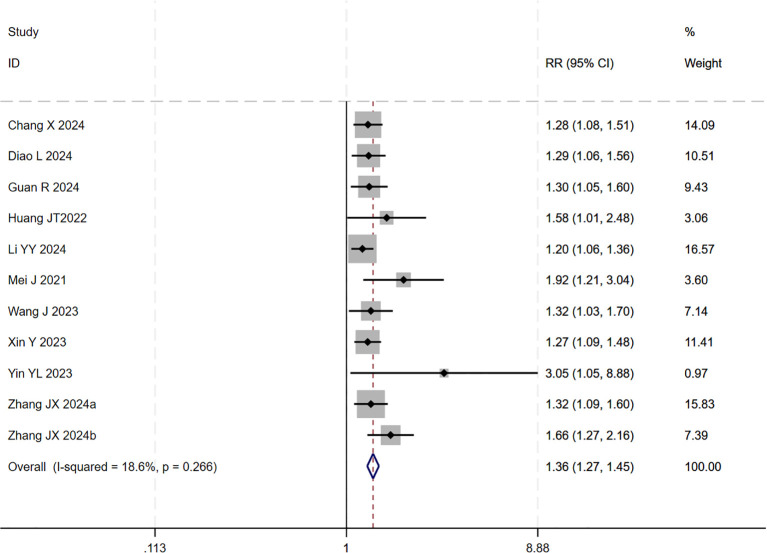
The forest plot demonstrates the effect of TACE/HAIC combined with targeted immunotherapy on DCR. Results show the combination significantly improves DCR compared to targeted immunotherapy alone. Squares represent RRs, horizontal lines indicate 95% CIs, and the diamond shows the pooled RR with its 95% CI. No significant heterogeneity was observed among studies (I² = 18.6%, p = 0.266). The overall pooled effect size was 1.36 (95% CI: 1.27, 1.45).

**Figure 8:**
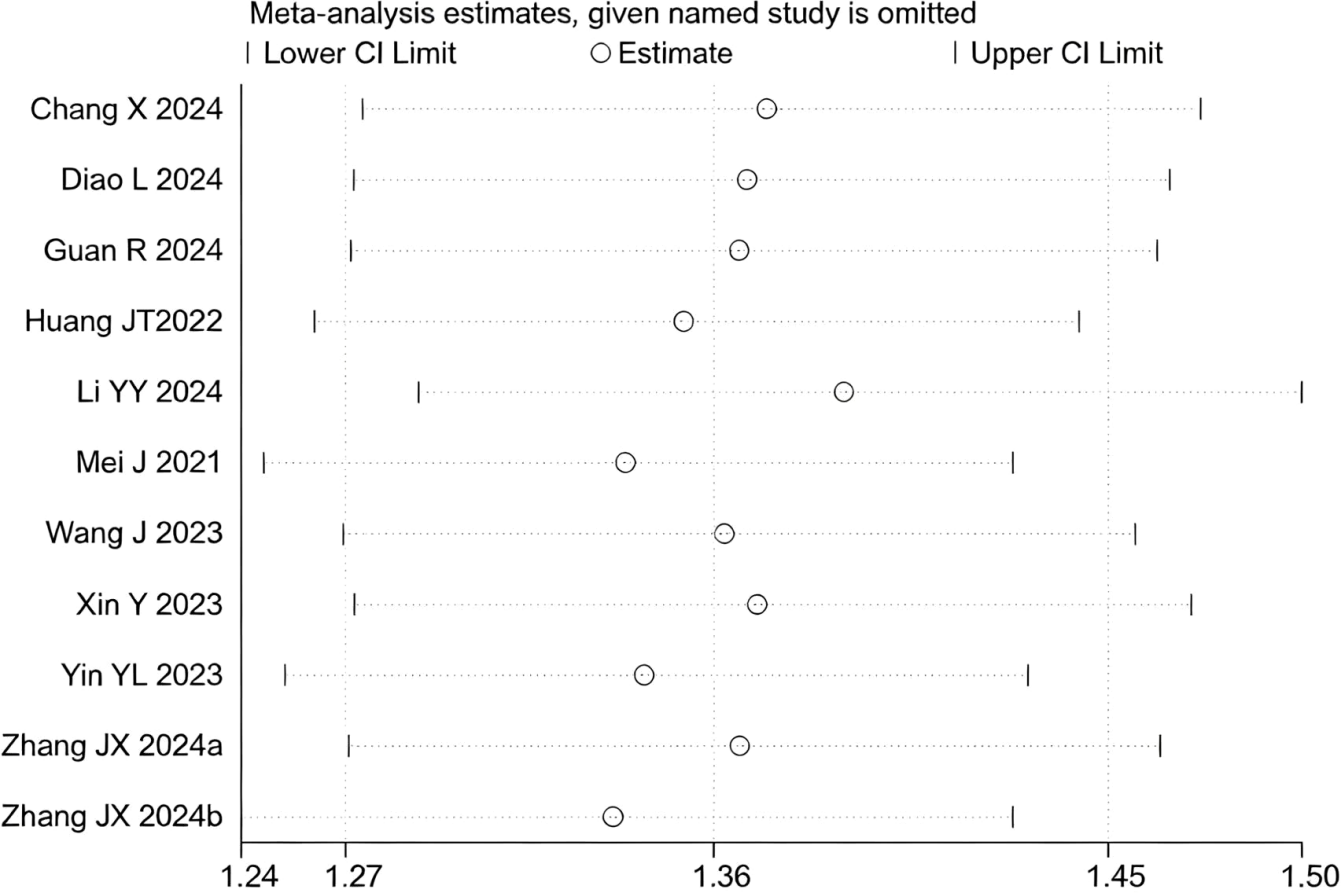
Heterogeneity test of the DCR results from the included studies. The sensitivity analysis revealed that there was no significant heterogeneity between the studies. Each circle on the vertical axis represents the exclusion of one experiment, and the short vertical lines at the two side ends represent the corresponding 95%CIs. From left to right, the three vertical lines represent the lower limit of the 95%CI, the mean, and the upper limit of the 95%CI for the overall effect.

12 studies (17–20, 22–29) reported data on the DCR. Initial pooled analysis revealed significant heterogeneity among these studies. To ensure the accuracy and robustness of the findings, subgroup and sensitivity analyses were conducted, which identified the study by (24) as the primary source of heterogeneity. After excluding this study, heterogeneity testing indicated no residual heterogeneity among the remaining studies. The pooled effect size demonstrated that triple therapy significantly improved DCR compared to dual therapy (RR=1.36, 95% CI: 1.27 to 1.45, P < 0.001) (**Figure 7**). Sensitivity analysis did not identify any additional studies that significantly influenced the results (**Figure 8**).

**Figure 9:**
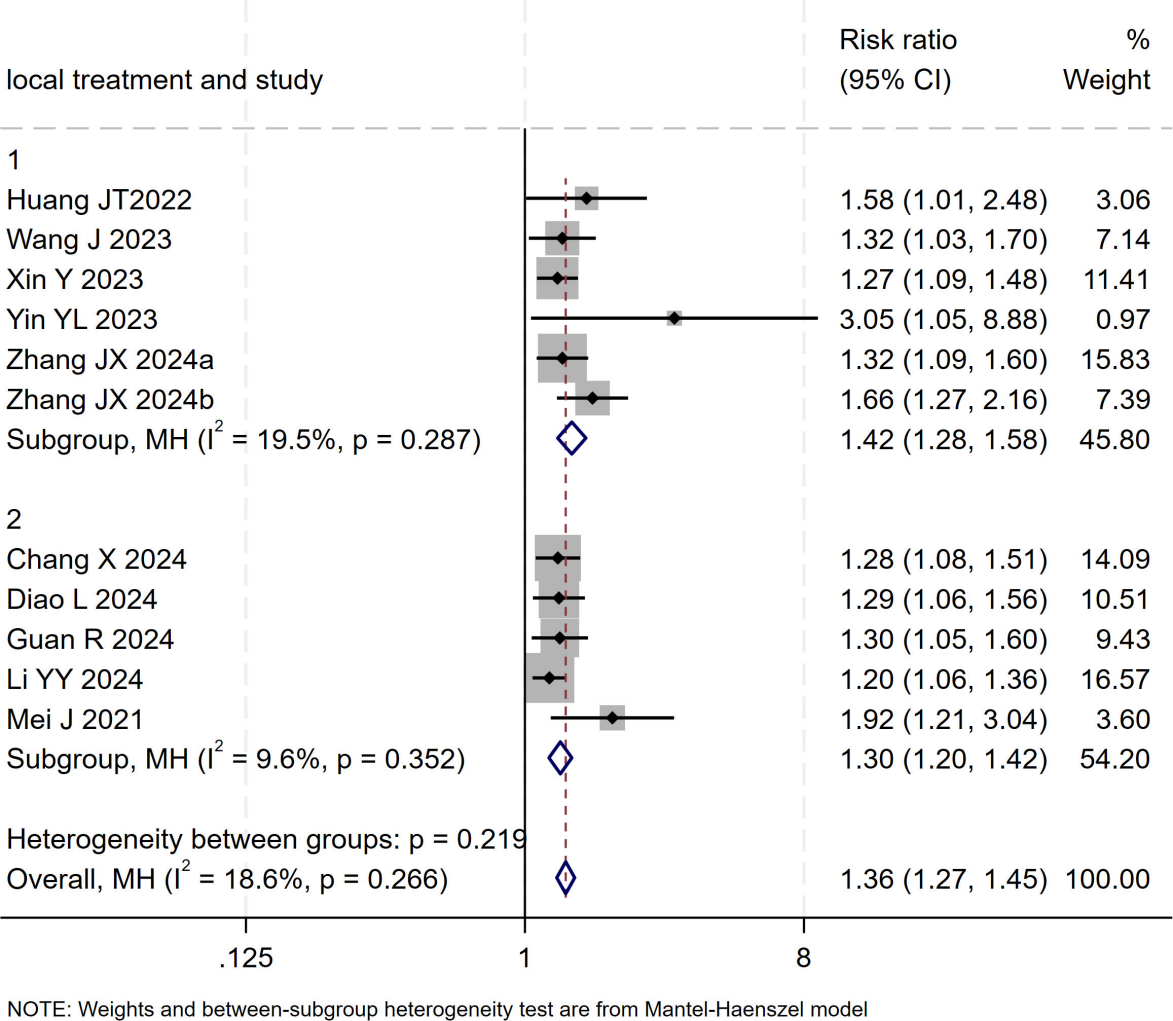
The forest plot presents a subgroup analysis of DCR based on local therapy (TACE vs. HAIC). No significant difference in DCR was found between subgroups (p=0.219). Squares represent RRs, horizontal lines show 95% CIs, and diamonds indicate pooled RRs. The TACE subgroup (RR=1.42, 1.28-1.58) and HAIC subgroup (RR=1.30, 1.20-1.42) both showed minimal heterogeneity (I²=19.5% and 9.6%). The overall pooled RR was 1.36 (1.27-1.45) with low heterogeneity (I²=18.6%, p=0.266).

Subgroup analysis based on the type of locoregional therapy (TACE vs. HAIC) was subsequently performed. The results indicated that triple therapy significantly improved DCR regardless of the locoregional approach used (P < 0.001 for both subgroups). The test for interaction between subgroups showed no statistically significant difference (p = 0.266), suggesting that the choice of locoregional modality did not substantially affect the overall DCR benefit of triple therapy (**Figure 9**).

Publication bias was assessed using funnel plot visualization and Egger’s test. Egger’s test result (p = 0.000 < 0.05) indicated the presence of potential publication bias among the 11 included studies. The trim-and-fill method was applied to adjust for the observed funnel plot asymmetry. The yellow dots represent the estimated effect sizes of potentially missing studies; the analysis suggested that incorporating approximately five additional studies with comparable results would be required to achieve symmetry and eliminate the observed publication bias (**Figure S4**).

### 3.6 ORR

13 studies (17–29) reported data on the ORR. The initial pooled analysis indicated moderate heterogeneity among the studies. Subgroup and sensitivity analyses identified the study by (26) as the primary source of this heterogeneity. After its exclusion, heterogeneity was significantly reduced and was no longer statistically significant, while the pooled result remained robust, demonstrating that triple therapy significantly improved the ORR.

**Figure 10:**
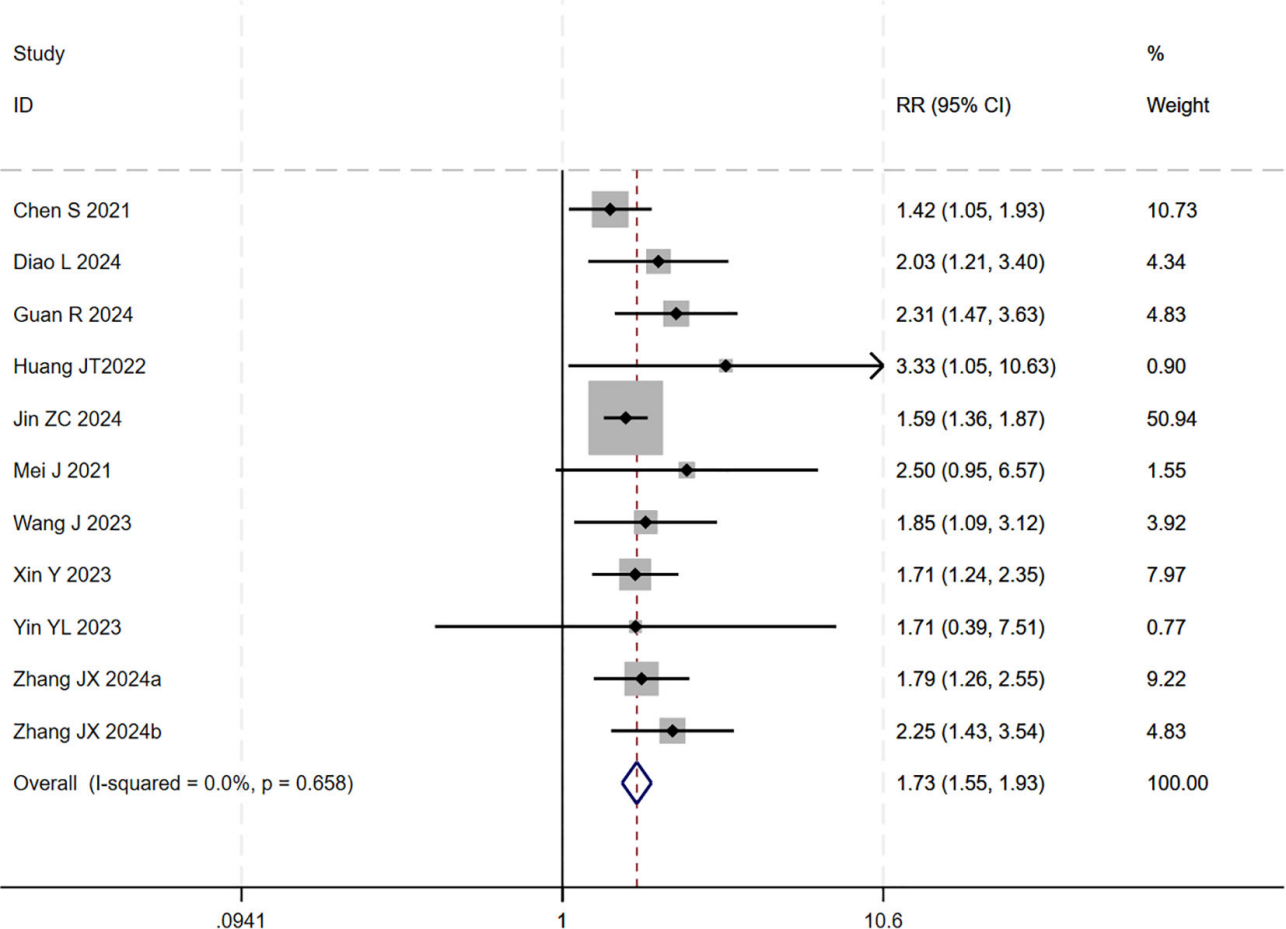
The forest plot demonstrates the effect of TACE/HAIC combined with targeted immunotherapy on ORR. Results indicate the combination significantly improves ORR compared to targeted immunotherapy alone. Squares represent risk ratios, horizontal lines indicate 95% CIs, and the diamond shows the pooled risk ratio with its 95% CI. No significant heterogeneity was observed among studies (I² = 0.0%, p = 0.658). The overall pooled effect size was 1.73 (95% CI: 1.55, 1.93).

**Figure 11:**
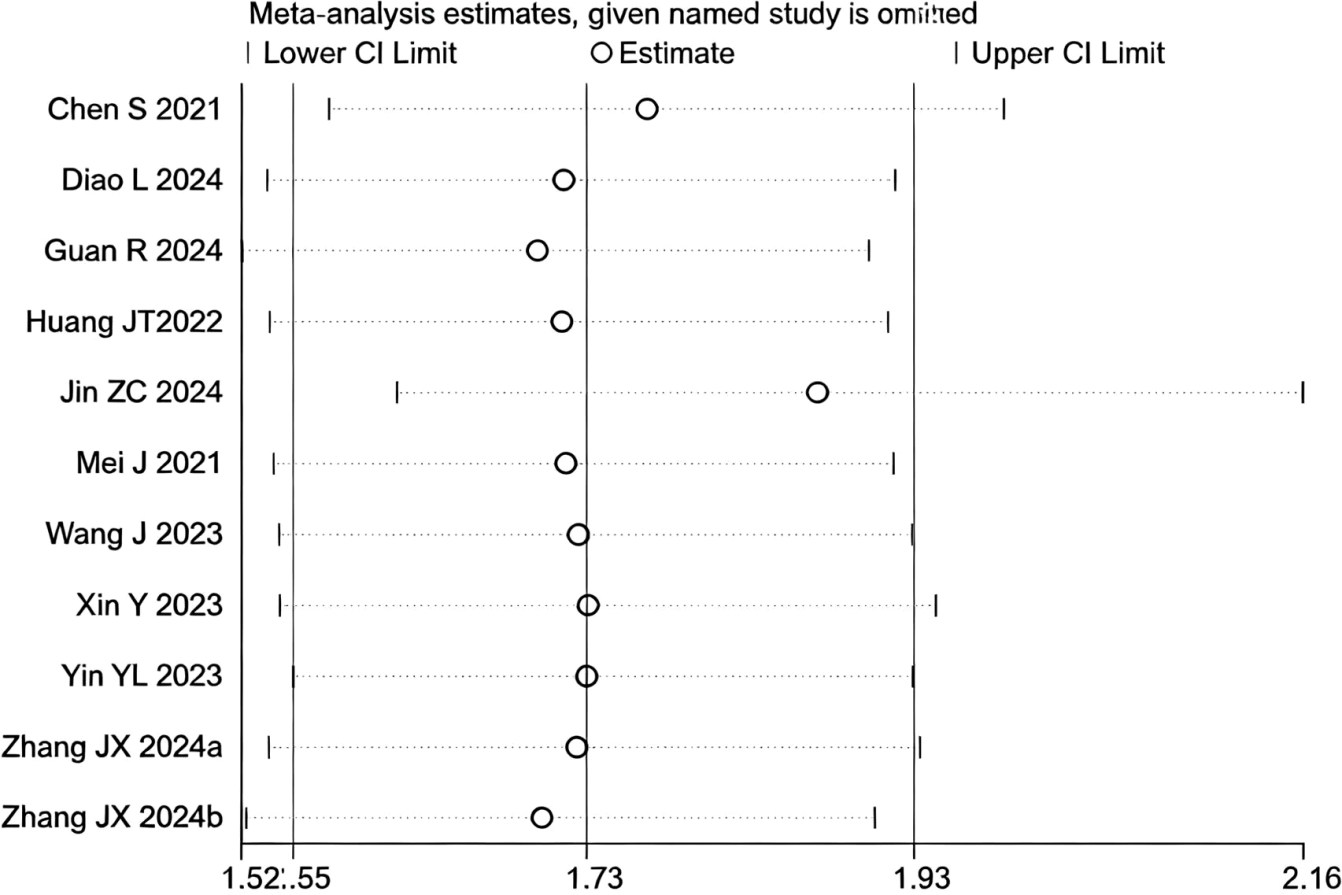
Heterogeneity test of the ORR results from the included studies. The sensitivity analysis revealed that there was no significant heterogeneity between the studies. Each circle on the vertical axis represents the exclusion of one experiment, and the short vertical lines at the two side ends represent the corresponding 95%CIs. From left to right, the three vertical lines represent the lower limit of the 95%CI, the mean, and the upper limit of the 95%CI for the overall effect.

However, upon subsequent subgroup analysis, significant heterogeneity was observed within the HAIC subgroup. Further sensitivity analysis specific to this subgroup identified the study by (29) as the main contributor to its heterogeneity. After the sequential removal of these studies, the final pooled analysis of 11 studies yielded no significant heterogeneity (I² = 0.0%, p = 0.66) and provided a more precise effect estimate: triple therapy significantly improved the ORR (RR=1.73, 95% CI: 1.55-1.93, P < 0.001) (**Figure 10**). Sensitivity analysis did not identify any other studies with a significant influence on the results (**Figure 11**).

**Figure 12:**
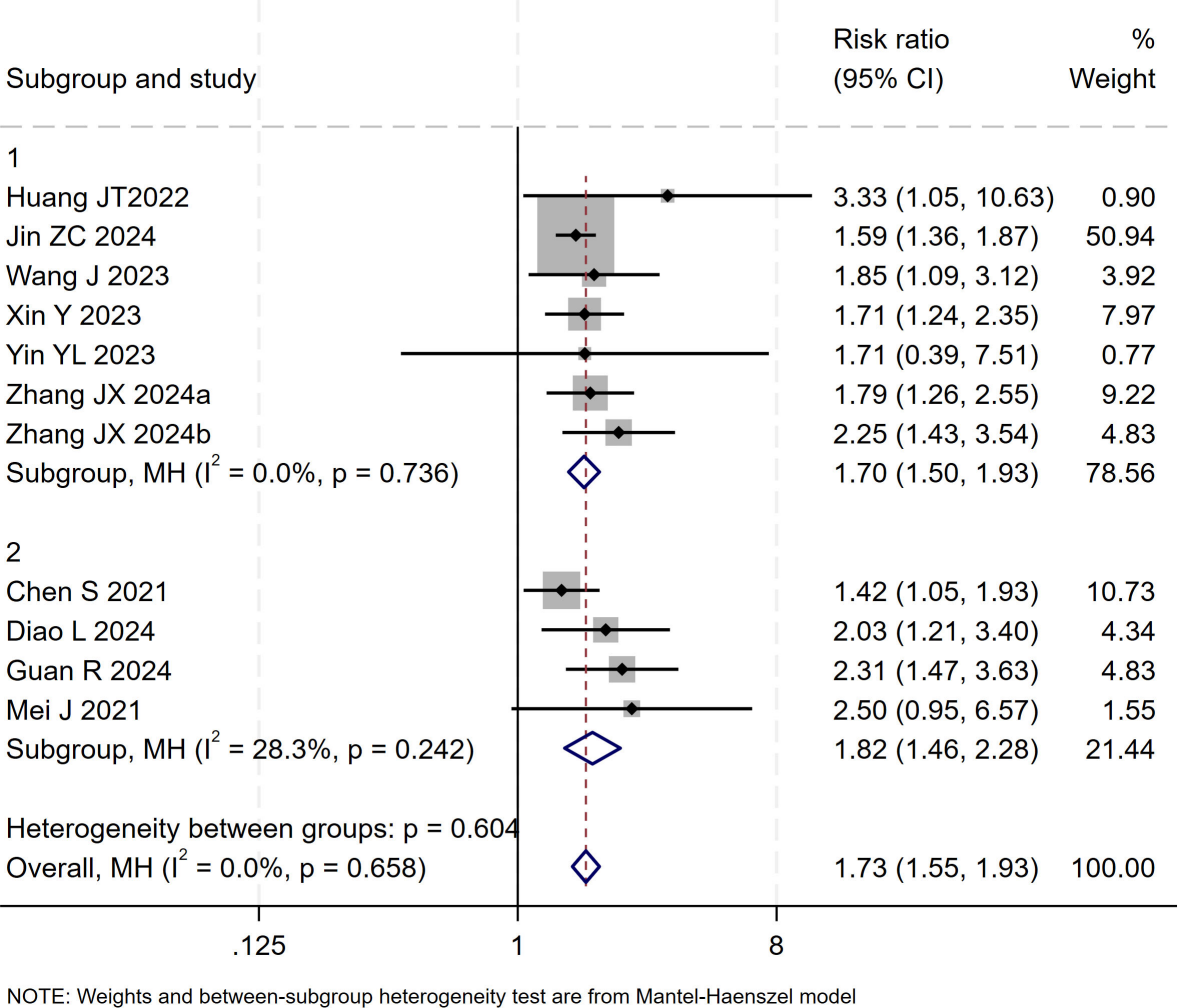
The forest plot presents a subgroup analysis of ORR by local therapy (TACE vs. HAIC). No significant difference in ORR was observed between subgroups (p=0.604). Squares represent RRs, horizontal lines show 95% CIs, and diamonds indicate pooled RRs. The TACE subgroup showed a pooled RR of 1.70 (1.50-1.93) with no heterogeneity (I²=0.0%), while the HAIC subgroup had a pooled RR of 1.82 (1.46-2.28) with low heterogeneity (I²=28.3%). The overall pooled RR was 1.73 (1.55-1.93) with no significant heterogeneity (I²=0.0%, p=0.658).

The test for interaction between subgroups based on the type of locoregional therapy showed no statistically significant difference in efficacy between TACE and HAIC (P for interaction = 0.60), suggesting that the choice of locoregional modality did not substantially affect the ORR benefit of the triple therapy (**Figure 12**).

Publication bias was assessed using a funnel plot and Egger’s test. The result of Egger’s test (p = 0.017 < 0.05) indicated the presence of potential publication bias among the 11 included studies. The trim-and-fill method was applied to adjust for the observed funnel plot asymmetry. The yellow dots represent the estimated effect sizes of potentially missing studies; the analysis suggested that incorporating approximately five additional studies with comparable results would be required to achieve symmetry and eliminate the observed publication bias (**Figure S5**).

### 3.7 Treatment-related AEs

All studies reported grade 1–2 AEs data, and one study reported grade 5 treatment-related AEs, with two cases appearing in the experimental group and one case in the control group, with no significant difference between the two groups(RR=1.44 [95%CI:0.15-15.87], p=1>0.05). The incidence rates of any grade AEs were 16.1% and 12.1% in the “triplet” and “binary” therapy groups, respectively. Regarding the incidence of AEs of any grade, there were significant differences between the two groups (RR 1.38 [95%CI 1.14, 1.67], P=0.00) and significant heterogeneity (I²=92.69%, P=0.00). The incidence rates of AEs of grade 3 or greater were 3.2% and 2.1%, which were statistically different (RR 1.48 [95%CI 1.20, 1.80], P=0.00), with significant heterogeneity (I²=54.87%, P=0.00).

The detailed AEs are presented in **Table 3**. The most common AEs included hypertension, fatigue, hand–foot–skin syndrome, diarrhea, decreased appetite, abdominal pain, fever, increased alanine aminotransferase (ALT), increased aspartate aminotransferase (AST), increased bilirubin, and thrombocytopenia. The incidence rates of fever, increases in AST and ALT in any grade or grade 3–5 AEs were higher in the experimental group than in the control group. The incidence rates of abdominal pain, nausea, vomiting, and other rare AEs were significantly higher in the experimental group than in the control group. No significant difference was found in the incidence of grade 3–5 AEs.

**Table 3:** Details of AEs.

Adverse events	All grade		Grade 3-5	
	RR (95% CI)	p	RR (95% CI)	p
Ascites	0.60 (0.32, 1.12)	0.12	–	–
Abdominal pain	2.27 (1.36, 3.78)	0.00	3.06(0.33,28.50)	0.32
Constipation	0.94 (0.24, 3.63)	0.93	–	–
Cough	1.25 (0.50, 3.09)	0.64	–	–
Decreased appetite	1.51 (0.98, 2.31)	0.06	1.42(0.68,2.94)	0.35
Diarrhea	1.06 (0.89, 1.25)	0.49	0.78(0.44,1.38)	0.40
Edema peripheral	1.67 (0.43, 6.48)	0.46	–	–
Fatigue	1.11(0.92, 1.35)	0.25	1.11(0.60,2.08)	0.73
Gastrointestinal hemorrhage	0.72 (0.40, 1.28)	0.27	–	–
Hand–foot skin reactions	1.16(0.99, 1.36)	0.06	1.31(1.14,1.51)	0.19
Hypertension	1.06 (0.95, 1.18)	0.28	1.08(0.87,1.32)	0.42
Hyperthyroidism	1.28 (0.63, 2.63)	0.49	–	–
Hypothyroidism	0.99(0.77,1.27)	0.94	1.82(0.61,5.42)	0.28
Immune-related hepatitis	1.52 (0.40, 5.75)	0.54	–	–
Pneumonitis	1.07 (0.66, 1.73)	0.78	1.25(0.26,6.08)	0.85
Immune-related hypophysitis	2.03 (0.30, 13.60)	0.47	–	–
Oral or anal ulcer	0.69 (0.33, 1.44)	0.34	–	–
Pyrexia	2.63 (1.52, 4.57)	0.00	1.79(1.05,3.03)	0.03
Pruritus	0.73 (0.44, 1.23)	0.24	1.16(0.23,5.99)	0.86
Proteinuria	1.05 (0.81, 1.34)	0.70	1.36(0.70,1.63)	0.36
Pain	2.32(0.99,5.42)	0.05	2.11(0.77,5.81)	0.14
Nausea	1.84 (1.31, 2.58)	0.00	2.01(0.41,9.68)	0.38
Vomiting	2.48 (1.32, 4.66)	0.00	2.18(0.77,6.17)	0.14
Rash	0.98 (0.79, 1.21)	0.85	0.95(0.46,1.97)	0.89
RCCEP	1.17 (0.84, 1.63)	0.36	1.12(0.51,2.48)	0.61
Weight decrease	1.18 (0.78, 1.78)	0.42	–	–
Elevated bilirubin	1.07 (0.88, 1.30)	0.48	0.97(0.41,2.27)	0.95
ElevatedGGT	1.27 (0.95, 1.71)	0.11	–	–
Increased AST	1.50 (1.13, 2.01)	0.01	1.61(1.06,2.45)	0.03
Increased ALT	1.51 (1.12, 2.03)	0.01	2.13(1.17,3.89)	0.01
Increased creatinine level	1.22 (0.92, 1.68)	0.20	0.68(0.19,2.38)	0.55
Increased serum uric acid level	1.36 (0.90,1.64)	0.65	–	–
Anaemia	1.02 (0.75, 1.39)	0.90	2.07(0.34,11.80)	0.44
Neutropenia	1.89 (0.82, 4.35)	0.13	1.35(0.79,2.32)	0.48
Leukopenia	1.17 (0.94, 1.46)	0.16	1.45(0.52,3.97)	0.67
Thrombocytopenia	1.17 (0.77, 1.78)	0.45	0.97(0.63,1.48)	0.88
Hypoalbuminemia	1.08 (0.84, 1.40)	0.56	1.60(0.65,3.93)	0.31
Others	1.86(1.36, 2.53)	0.00	1.60(0.54,4.71)	0.40

RCCEP, reactive cutaneous capillary endothelial proliferation; ALT, alanine transaminase; AST, aspartate transaminase; GGT, g-glutamyl transpeptidase.

## 4 Discussion

This meta-analysis investigated the efficacy and safety of triple therapy (TACE/HAIC combined with targeted and immunotherapy) in patients with hepatocellular carcinoma. The results demonstrated that the combination significantly improved OS, PFS, DCR, and ORR. Although the overall incidence of AEs was statistically significantly increased, no elevated risk of grade 5 serious AEs was observed, supporting a favorable benefit-risk profile for clinical application.

HCC ranks among the most common malignant tumors worldwide and is a leading cause of cancer-related mortality (30). TACE and HAIC are widely adopted in clinical practice as well-established locoregional interventions for HCC. However, the efficacy of TACE or HAIC alone is considerably limited. For instance, in patients with intermediate to advanced-stage disease treated with TACE, the 1-year survival rate is approximately 54%, while the 5-year survival rate drops to only 16% (6). Similarly, HAIC monotherapy demonstrates limited efficacy in controlling micrometastatic lesions (31–34). Targeted agents [e.g., sorafenib, with a median OS of only 6.5-10.7 months (35)] and PD-1/PD-L1 inhibitor monotherapies also present significant shortcomings, including primary resistance rates as high as 30%-50% and the frequent emergence of secondary resistance within 6–12 months of treatment (36, 37). This meta-analysis is the first to systematically elucidate the potential value of the triple therapy in HCC management. Within the current clinical context where atezolizumab plus bevacizumab is recommended as a first-line treatment for HCC (35, 38), the triple therapy demonstrates significant additional improvements in OS and PFS, positioning it as a promising new candidate standard of care. This approach may be particularly beneficial for patients with high localized tumor burden or those at risk of treatment resistance.

Our study clearly demonstrates that TACE/HAIC combined with targeted and immunotherapy prolongs OS and PFS, while also improving the DCR and ORR in patients with hepatocellular carcinoma. These findings are entirely consistent with the conclusions of the 13 studies included in our analysis (17–29). This favorable therapeutic effect stems from a synergistic interplay among the treatment modalities, the core mechanism of which can be summarized as a closed-loop process of “local debulking – targeted regulation – immune clearance.” The arterial blood supply dependency of HCC provides a critical therapeutic target for locoregional interventions. TACE achieves a dual blockade of tumor nutrient supply through “chemotherapeutic drug infusion plus vascular embolization, “ whereas HAIC maintains high local concentrations of chemotherapeutic agents via sustained arterial infusion; both directly kill tumor cells. Targeted therapy can overcome intrinsic tumor resistance by inhibiting signaling pathways such as MAPK, WNT-β-catenin, CDK4-CDK6, or PTEN, while also suppressing local therapy-induced VEGF expression, thereby reducing tumor angiogenesis (39). More importantly, locoregional therapies promote the release of tumor neoantigens and upregulate pro-inflammatory cytokine levels. Targeted therapy, in turn, improves immune cell infiltration through vascular normalization. These actions synergistically activate the body’s immune response, facilitating the transition from an immunologically “cold” to a “hot” tumor microenvironment and significantly enhancing the antitumor efficacy of immune checkpoint inhibitors (40, 41). Furthermore, locoregional therapy modifies the tumor microenvironment, for instance, by promoting the release of VEGF and hypoxia-inducible factor-1α (HIF-1α), which further potentiates the effect of targeted agents. Concurrently, these molecular changes can activate dendritic cells and recruit them into the tumor microenvironment, shifting it from an immunosuppressive state to one favorable for the action of immune checkpoint inhibitors (42), thereby creating multidimensional synergistic effects.

It is noteworthy that in the comparison of AEs, although the overall incidence of AEs was higher in the triple-therapy group compared to the dual-therapy group, there was no significant difference in the incidence of serious grade 5 treatment-related AEs between the two. Only a few specific AEs (such as gastrointestinal reactions and mild liver function abnormalities) showed a statistically significant increase. This finding is supported by the study by Xiao Y et al. (43), which evaluated the impact of triple therapy (HAIC combined with TKIs and a PD-1 inhibitor) in patients with unresectable HCC (uHCC) stratified by Child-Pugh grade into CPA (grade A) and CPB (grade B) groups. The results indicated that patients with better liver function (CPA group) achieved superior treatment outcomes, while the CPB group was more susceptible to AEs—a difference potentially attributable to poorer liver functional reserve and impaired drug metabolism leading to higher systemic drug levels in the CPB group. This conclusion supports the view in (21) of a potential cumulative effect of adverse reactions from combination therapy. Divergent findings from other studies (17–29), which reported no significant difference in AE incidence, may be due to heterogeneity in treatment protocols or limited sample sizes. These methodological variations likely account for the difference with our meta-analysis results. This study conducted a subgroup analysis based on the core mechanistic difference between TACE, which employs combined chemoinfusion and embolization, and HAIC, which uses sustained arterial infusion for local enrichment, to systematically compare their respective outcomes (OS, PFS, DCR, ORR) within combination therapy. The results indicated no statistically significant differences in these core efficacy endpoints between the two subgroups. However, this finding contrasts with conclusions from several previous studies. Yu B et al. (44), in a large-sample retrospective study, explicitly stated that the efficacy of HAIC combined with targeted and immunotherapy was comprehensively superior to the TACE-based combination: after PSM, the HAIC group showed significantly prolonged OS (not reached vs. 12.4 months) and PFS (14.5 months vs. 6.8 months), alongside overwhelming advantages in ORR (52.5% vs. 15.9%) and DCR (79.7% vs. 40.1%). The researchers proposed a key mechanistic insight: continuous arterial infusion in HAIC establishes a sustained, high-concentration chemotherapeutic milieu within the tumor. This pharmacokinetic profile acts in synergy with the anti-angiogenic effects of TKIs and the immune-promoting actions of anti-PD-1 antibodies, collectively establishing a triple synergy of chemotherapeutic cytotoxicity, targeted anti-angiogenesis, and immune-mediated clearance. In contrast, while TACE’s embolization rapidly blocks tumor blood supply, it induces hypoxic stress in the TME, activating the HIF-1α signaling pathway and upregulating pro-angiogenic factors like VEGF and PDGF, consequently driving tumor neovascularization and local recurrence, ultimately undermining the long-term benefit of the combination therapy. Notably, even in the context of single locoregional therapy, HAIC has been demonstrated to yield superior tumor control and survival benefits compared to TACE (45). Another study by Long T et al. (46) offered a more nuanced perspective: although HAIC combined with targeted and immunotherapy did not significantly improve overall OS, it provided pronounced PFS benefits in high-risk subgroups with large tumor volume (>10cm) or vascular invasion (median PFS 15.0 months vs. 6.4 months, P=0.028). The core reason is that HAIC’s continuous infusion mode ensures adequate local drug concentration in intrahepatic lesions, effectively clearing micro-metastases in large tumors and areas with vascular invasion. The authors also noted that both TACE and HAIC, as locoregional therapies, can induce TME hypoxia and subsequent VEGF upregulation through different pathways; this common pathological change might partially offset the differences in local drug delivery efficiency, potentially leading to the lack of a significant overall efficacy difference. The discrepancy between our findings and previous conclusions may stem from multiple factors: first, the sample size for the subgroup analysis in this study was relatively limited, and further reduction upon stratification may have compromised statistical power; second, potential heterogeneity in baseline patient characteristics, such as uneven distribution of BCLC stages, liver function reserve (ALBI grade), and tumor burden (presence of PVTT, extrahepatic metastasis)—key prognostic factors—could confound the direct association between treatment modality and efficacy; third, variations in drug selection within the combination regimens (e.g., types of TKIs, specific PD-1 inhibitors) and individualized adjustments to treatment cycles might also influence the final outcomes.

The strengths of this study lie in its strict adherence to methodological guidelines for systematic reviews and meta-analyses—such as comprehensive search strategies, explicit inclusion and exclusion criteria, and risk of bias assessment—and the inclusion of a relatively large number of studies, which collectively enhance the accuracy and reliability of the findings. Nevertheless, several limitations should be acknowledged. First, as with other meta-analyses, considerable inherent heterogeneity exists among the included studies. Variations in patient baseline characteristics (e.g., age, etiology), disease stage, treatment protocols (e.g., cycles of locoregional therapy, types of targeted/immunotherapeutic agents), and efficacy evaluation criteria (Response Evaluation Criteria in Solid Tumours, RECIST vs. Modified Response Evaluation Criteria in Solid Tumors, mRECIST) may affect the robustness of the results, necessitating further validation through large-scale randomized controlled trials (RCTs). Second, all included studies were conducted in China, limiting the generalizability of the findings to other regions and ethnic groups. Third, the exclusive inclusion of retrospective studies introduces potential biases in patient selection, data collection, and outcome reporting, which may compromise internal validity. Finally, due to limited data availability from the original studies, subgroup analyses could only be performed based on the type of locoregional therapy and were unable to stratify by key factors such as disease stage, patient age, or presence of vascular invasion. Important outcome measures such as quality of life were also unavailable. These issues warrant further investigation in future studies.

## 5 Conclusions

In summary, this meta-analysis provides preliminary evidence-based support for the clinical application of TACE/HAIC combined with targeted and immunotherapy in patients with intermediate to advanced hepatocellular carcinoma, demonstrating that this triple therapy significantly improves survival outcomes and tumor response with a manageable safety profile. However, given the limitations of this study, further large-scale, multicenter, prospective randomized controlled trials are warranted to validate the reliability of these findings, identify the patient subgroups most likely to benefit, and optimize treatment protocols, thereby providing higher-level evidence for individualized management of intermediate to advanced HCC.

## Data Availability

The original contributions presented in the study are included in the article/**Supplementary Material**. Further inquiries can be directed to the corresponding authors.
