# Supplementary material for: Efficacy and safety of TACE/HAIC combined with targeted immunotherapy versus targeted immunotherapy for advanced hepatocellular carcinoma: a meta-analysis
Source: Front Oncol. 2025 Nov 19;15:1669460. doi: 10.3389/fonc.2025.1669460 (PMC12673508; doi:10.3389/fonc.2025.1669460)
Supplement: Supplementary file 1 [file DataSheet1.docx]

**Supplementary figures**

Supplementary figures


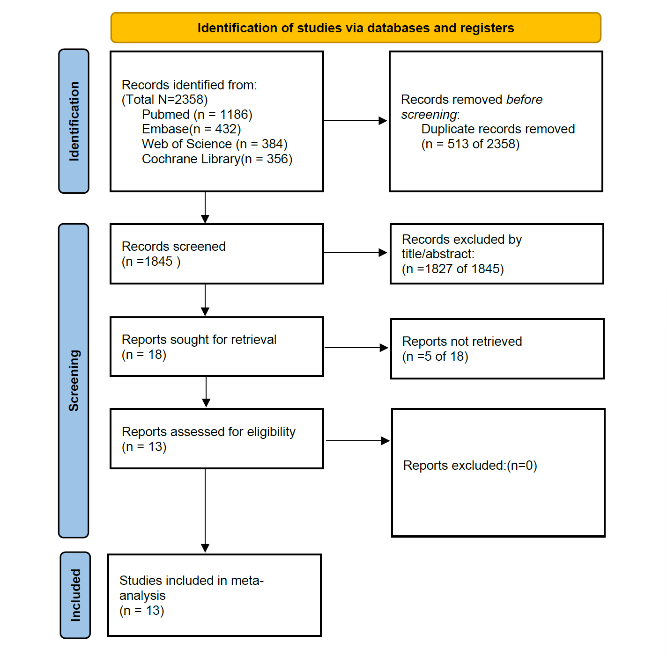


Figure.S 1. Study screening process for inclusion in the meta-analysis.


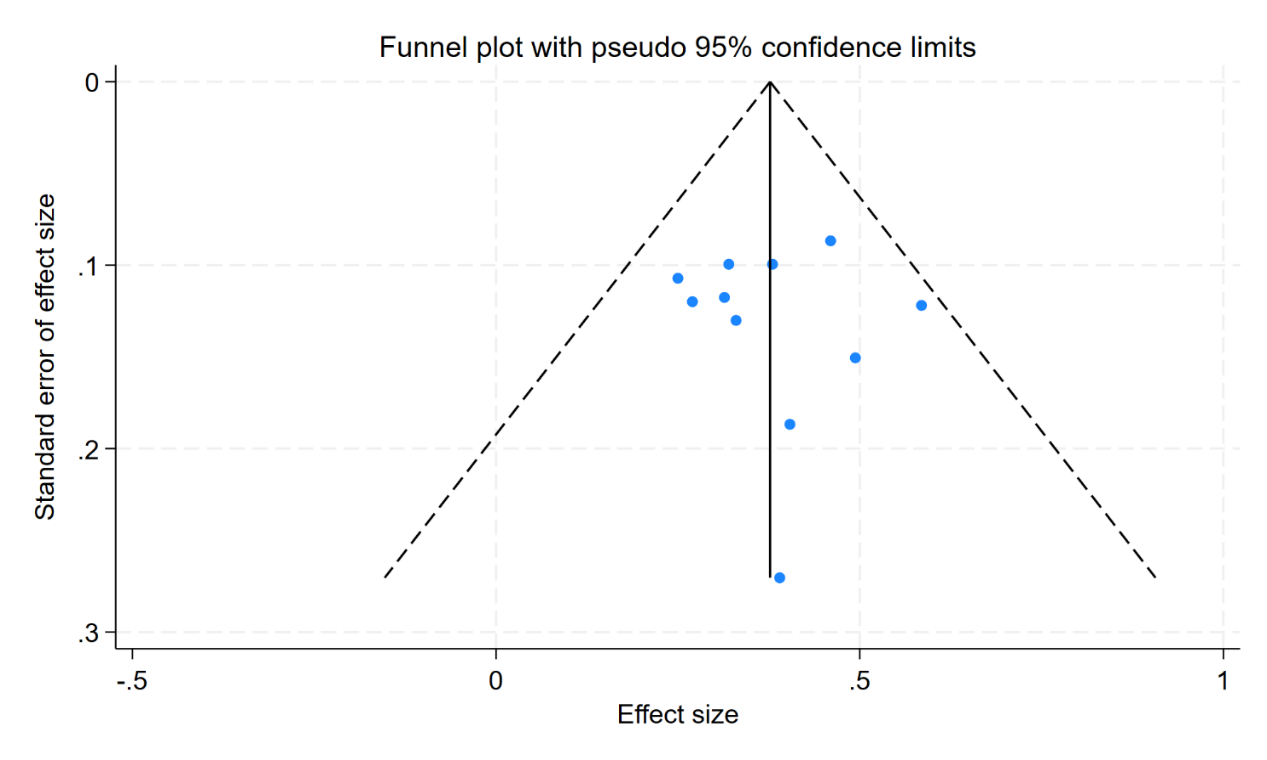


Figure.S 2. This is a funnel plot used to assess OS publication bias. The horizontal axis is Effect size, and the vertical axis is Standard error of effect size. The black dashed lines represent Pseudo 95% confidence limits, and the blue dots represent the included studies. The distribution of study points within the pseudo confidence intervals can be used to preliminarily determine the presence of publication bias.


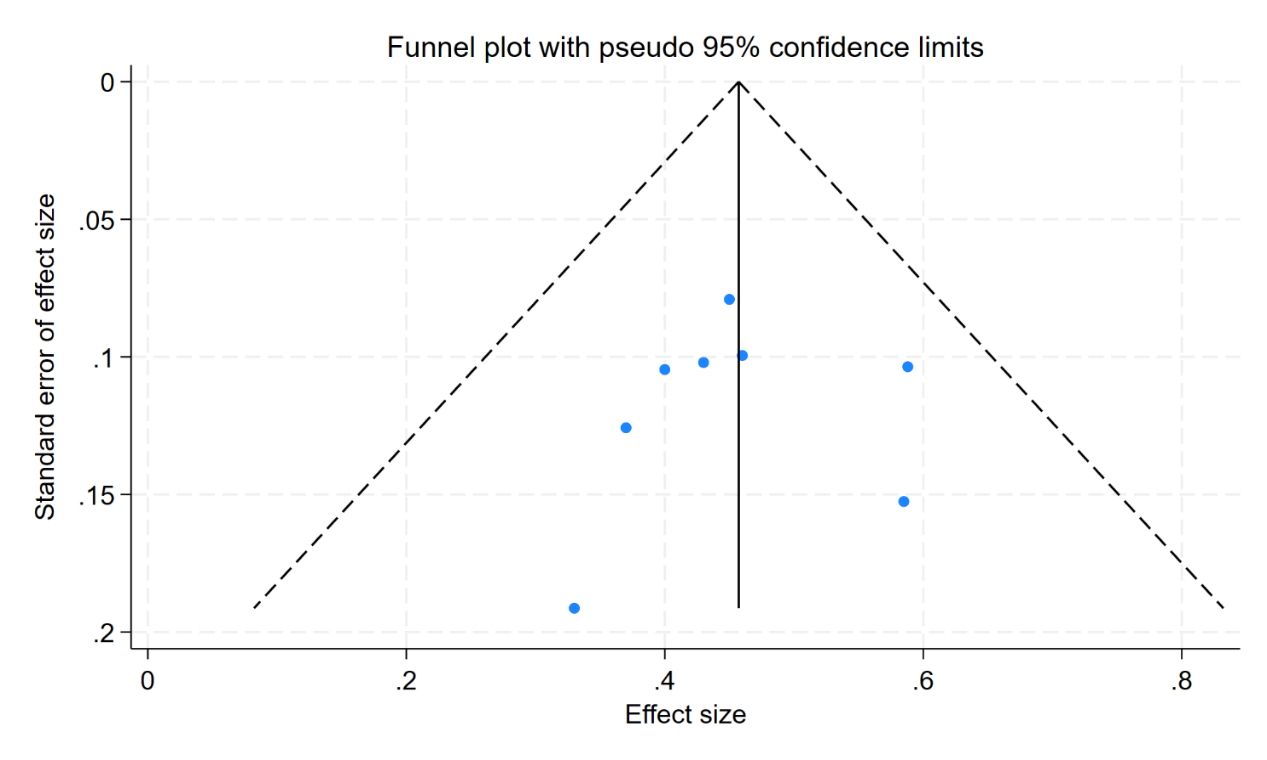


Figure.S 3. This is a funnel plot used to assess PFS publication bias. The horizontal axis is Effect size, and the vertical axis is Standard error of effect size. The black dashed lines represent Pseudo 95% confidence limits, and the blue dots represent the included studies. The distribution of study points within the pseudo confidence intervals can be used to preliminarily determine the presence of publication bias.


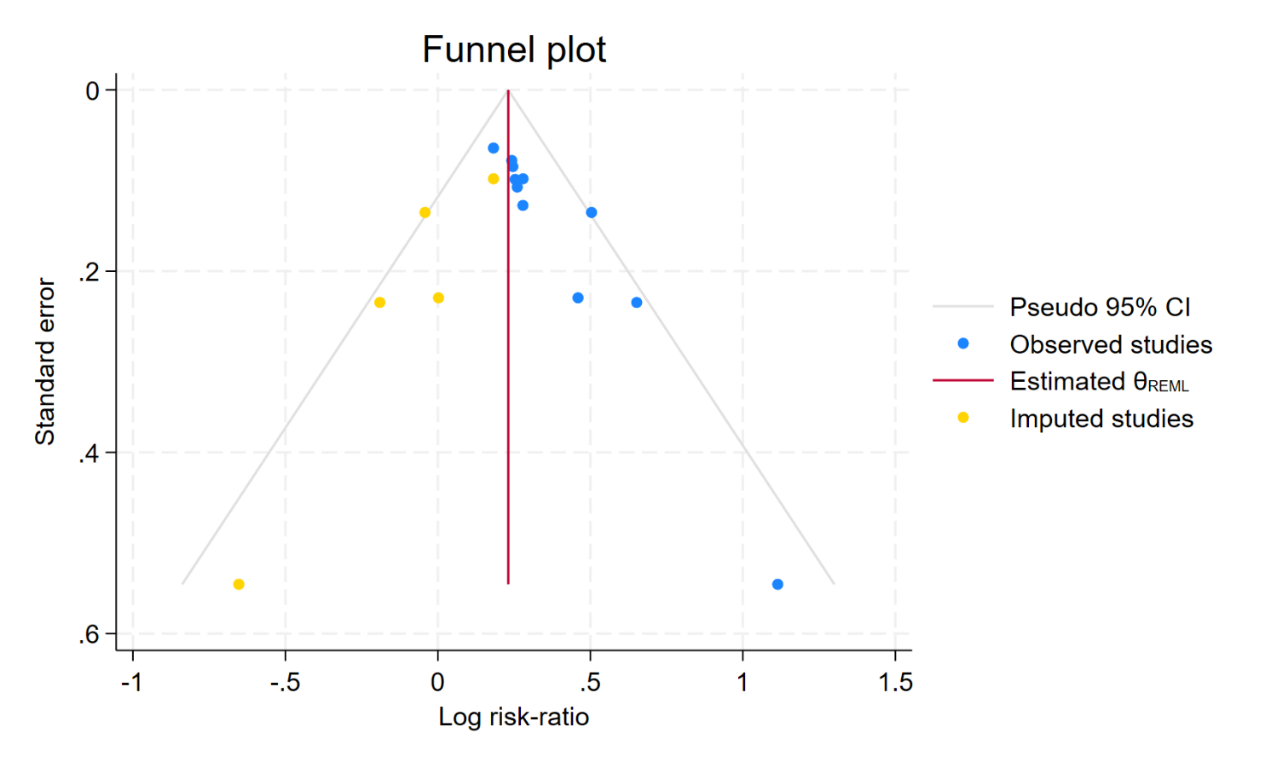


Figure.S 4 This is a funnel plot after the trim-and-fill method, used to assess DCR publication bias. The horizontal axis is Log risk-ratio, and the vertical axis is Standard error. The gray area represents Pseudo 95% CI; blue dots represent Observed studies; the red vertical line represents Estimated θ₍ₚₑₘₗ₎ (restricted maximum likelihood estimated effect size); yellow dots represent Imputed studies, which are simulated to represent potentially missing studies for evaluating publication bias.


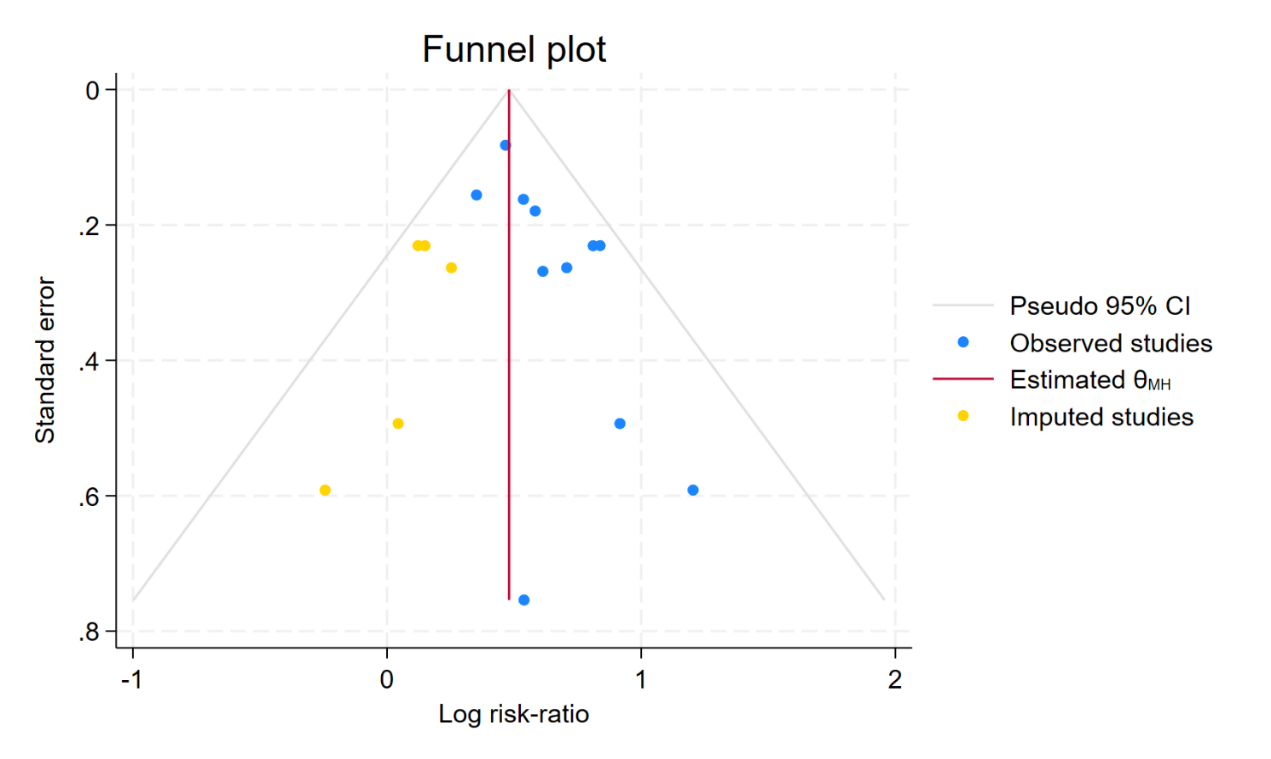


Figure.S 5 This is a funnel plot after the trim-and-fill method, used to assess ORR publication bias. The horizontal axis is Log risk-ratio, and the vertical axis is Standard error. The gray area represents Pseudo 95% CI; blue dots represent Observed studies; the red vertical line represents Estimated θ₍ₚₑₘₗ₎ (restricted maximum likelihood estimated effect size); yellow dots represent Imputed studies, which are simulated to represent potentially missing studies for evaluating publication bias.
